# Multidisciplinary recommendations for essential baseline functional and laboratory tests to facilitate early diagnosis and management of immune-related adverse events among cancer patients

**DOI:** 10.1007/s00262-023-03436-0

**Published:** 2023-04-05

**Authors:** Berna C. Özdemir, Cristina Espinosa da Silva, Dimitri Arangalage, Pierre Monney, Sabina A. Guler, Uyen Huynh-Do, Guido Stirnimann, Lucia Possamai, Roman Trepp, Robert Hoepner, Anke Salmen, Camille L. Gerard, Petr Hruz, Lisa Christ, Sacha I. Rothschild

**Affiliations:** 1 Department of Medical Oncology, Inselspital, Bern University Hospital, University of Bern, Bern, Switzerland; 2 Herbert Wertheim School of Public Health and Human Longevity Science, University of California, San Diego, La Jolla, CA USA; 3 Division of Epidemiology and Biostatistics, School of Public Health, San Diego State University, San Diego, USA; 4 Department of Cardiology, INSERM U1148, Bichat Hospital, University of Paris, Paris, France; 5 Department of Cardiology, Lausanne University Hospital (CHUV), University of Lausanne, Lausanne, Switzerland; 6 Department of Pulmonary Medicine, Inselspital, Bern University Hospital, University of Bern, Bern, Switzerland; 7 Department of Nephrology and Hypertension, Inselspital, Bern University Hospital, University of Bern, Bern, Switzerland; 8 Department of Visceral Surgery and Medicine, Inselspital, Bern University Hospital, University of Bern, Bern, Switzerland; 9 Department of Metabolism, Digestion and Reproduction, Faculty of Medicine, Imperial College London, London, UK; 10 Department of Diabetes, Endocrinology, Nutritional Medicine and Metabolism (UDEM), Inselspital, Bern University Hospital, University of Bern, Bern, Switzerland; 11 Department of Neurology, Inselspital, Bern University Hospital, University of Bern, Bern, Switzerland; 12 Department of Oncology, Lausanne University Hospital (CHUV), University of Lausanne, Lausanne, Switzerland; 13 The Francis Crick Institute, London, UK; 14 Department of Gastroenterology, University Hospital Basel, Basel, Switzerland; 15 Department of Rheumatology and Immunology, Inselspital, Bern University Hospital, University of Bern, Bern, Switzerland; 16 Department of Medical Oncology, University Hospital Basel, Basel, Switzerland; 17 Department Internal Medicine, Center for Oncology and Hematology, Cantonal Hospital Baden, Baden, Switzerland

**Keywords:** Immune checkpoint inhibitors, Minimal data set, Laboratory tests, Baseline evaluation, Diagnosis, Immune-related adverse events

## Abstract

Immune checkpoint inhibitors (ICIs) have fundamentally changed the treatment landscape of various cancers. While ICI treatments result in improved survival, quality of life and are cost-effective, the majority of patients experience at least one immune-related adverse event (irAE). Many of these side effects cause little discomfort or are asymptomatic; however, irAEs can affect any organ and are potentially life-threatening. Consequently, early diagnosis and appropriate treatment of irAEs are critical for optimizing long-term outcomes and quality of life in affected patients. Some irAEs are diagnosed according to typical symptoms, others by abnormal findings from diagnostic tests. While there are various guidelines addressing the management of irAEs, recommendations for the early recognition of irAEs as well as the optimal extent and frequency of laboratory tests are mostly lacking. In clinical practice, blood sampling is usually performed before each ICI administration (i.e., every 2–3 weeks), often for several months, representing a burden for patients as well as health care systems. In this report, we propose essential laboratory and functional tests to improve the early detection and management of irAEs and in cancer patients treated with ICIs. These multidisciplinary expert recommendations regarding essential laboratory and functional tests can be used to identify possible irAEs at an early time point, initiate appropriate interventions to improve patient outcomes, and reduce the burden of blood sampling during ICI treatment.

## Introduction

Since the approval of ipilimumab in 2011, immune checkpoint inhibitors (ICIs) are increasingly used in the standard-of-care for multiple cancer types. ICIs have changed the treatment paradigm in many cancer types and have improved survival in a subset of patients with advanced or metastatic cancers [1–3]. However, despite their clinical efficacy, ICIs can induce various immune-related adverse events (irAEs) limiting their use in some patients. While many irAEs are low grade, reversible, and managed with symptomatic treatment and withholding ICIs, some irAEs are high-grade and require definitive treatment interruption, immunosuppressive treatment and hospitalization, with some patients suffering from long-term residual impairment [4].

Reported hospitalization rates of irAEs ranged from 8.5 to 25%, with an increased risk in older individuals (> 65 years) and those undergoing combination therapy (i.e., anti-CTLA4 and anti-PD1) [5, 6]. Other studies in similar settings have found that ICI-treated patients with irAEs have a median hospital stay of 5–10 days [5, 7–9], with a readmission rate of 17–25% for other irAEs [7, 8] and a mortality rate of 8–13% [7, 9]. Moreover, about one-third of patients undergoing treatment with ICIs present to the emergency department at least once, and 18% of these visits are related to irAEs [10]. Notably, the incidence of irAEs in real-world settings seems higher compared to clinical trials [11], possibly due to the typically older and multimorbid patient population outside of clinical trials. There are currently limited data available on the effectiveness and tolerability of ICIs in elderly individuals and patients with poor performance status in clinical practice [12, 13]. The frequent reoccurrence of irAE when patients are rechallenged with ICIs additionally highlights the need for close monitoring throughout the duration of treatment [14].

Most irAEs occur during the first 3–4 months of treatment initiation, although delayed toxicity (defined as irAEs arising after one year of treatment) is also possible [15, 16]. In a pooled analysis of 23 phase II and III clinical trials that tested anti-PD1, anti-PDL1 alone, or anti-PDL1 in combination with anti-CTLA4 antibodies or other anticancer therapies, the pooled median time to onset of all-grade irAEs was between 2 and 15 weeks. Combination ICI treatments had a shorter time to onset than ICIs targeting PD1 or PDL1 alone [17]. Importantly, a prospective study found that very severe irAES occurred significantly earlier (median of 41 days of ICI initiation) compared to low-grade irAEs [18].

Given these findings, the early recognition and appropriate management of irAEs are essential to optimize long-term outcomes and quality of life. The diagnosis and treatment of irAEs typically require consultation by a specialist and specific laboratory tests, imaging, and functional tests. The interpretation of test results for suspected irAEs can be challenging if no baseline values are available, particularly in patients with comorbidities that limit specific organ function. In addition, the assessment of irAE occurrence, grading, and timing is highly variable among clinicians [19]. The thorough documentation of pre-treatment status (e.g., comorbidities) and baseline test results prior to initiation of ICIs can consequently facilitate irAE diagnosis. While there are various guidelines for the management of irAEs, expert [20] guidance is limited and outdated [21] regarding which laboratory and diagnostic tests should be performed before starting ICIs and during ICI treatment [21]. In absence of specific guidelines for clinicians, patients might undergo blood sampling before each ICI administration (i.e., every 2–3 weeks), for several years. This emphasizes the need for updated and more practical recommendations. In addition, the broadening indications for ICIs, the aging population, as well as the increasing use of ICIs in populations at risk (e.g., patients with preexisting autoimmune disease or chronic infections) will likely lead to clinicians being confronted with irAEs more often. Consequently, there is a critical need to propose a set of minimal baseline assessments which facilitate toxicity detection with the least burden for patients and health care systems.

We provide practical recommendations for essential baseline laboratory and functional tests compiled by a multidisciplinary team of experts for the systematic assessment of various organ systems before starting ICIs and at regular intervals during ICI treatment. If patients present with any abnormal baseline values, the frequency and extent of the blood and functional tests should be adapted on an individual basis.

A multidisciplinary panel composed of experts from medical oncology, cardiology, endocrinology, gastroenterology, hepatology, nephrology, neurology, pneumology, rheumatology, and public health who convened to develop the clinical recommendations. The experts met via teleconference and corresponded through e-mail. After agreement on the scope and objectives of the consensus manuscript, key topics were discussed in an iterative process until full agreement was reached. Typically authors had the last authority on discussion points pertaining to their specific area of expertise.

A scoping literature search was performed using the PubMed database to obtain key literature on ICI-related toxicity and laboratory tests published using ICI–specific terms combined with “irAEs, laboratory tests, functional tests, dataset, recommendations”. Consensus discussions were repeated after gathering new evidence, and specific articles to include in the manuscript were selected by the experts based on their methodological rigor and importance for the key objectives.

### Liver function

Clinically apparent liver toxicity due to ICI treatment is frequent. The risk for hepatotoxicity is higher with ICI combination treatment (13%) than with anti-PD1/PDL1 monotherapy (5%) and is dose-dependent [22–24]. Hepatitis is usually observed after 7–8 weeks of treatment, but an earlier manifestation is possible [25, 26]. Classical liver autoantibodies are usually not seen in ICI-induced hepatitis. The histological pattern of liver injury is variable. Lobular hepatitis was mainly observed in patients treated with anti-PD1/PDL1 and fibrin ring granulomas and endotheliitis seem to be a particular feature in anti-CTLA4 treated patients. Spotty necrosis or confluent necrosis are features of more severe cases, independent of the ICI used [27].

We recommend assessing baseline liver tests in two stages. Serum aspartate aminotransferase (AST), alanine aminotransferase (ALT), gamma-glutamyl transferase (GGT), alkaline phosphatase (ALP), and total bilirubin should be measured in all patients (Table 1). In case of elevated liver function tests on initial screening, potential preexisting conditions should be ruled out by targeted investigations and imaging to identify liver infiltration, metastatic disease, biliary obstruction, metabolic steatohepatitis (MASH), drug induced hepatitis (DILI), viral hepatitis, and autoimmune hepatitis. Liver tests should be performed prior to each ICI administration during the first 3 months and every 4–6 weeks thereafter.

**Table 1:** Essential laboratory and functional tests to evaluate before and during treatment with immune checkpoint inhibitors (ICIs)

	Prior to ICI treatment	During ICI treatment			
	Baseline	During the first 3 months		After the first 3 months	
		Every ICI administration	Every 4–6 weeks	Every 4–6 weeks	Every 3–6 months
Liver	AST, ALT, GGT, ALP, total bilirubin	AST, ALT, GGT, ALP, total bilirubin	–	AST, ALT, GGT ALP, total bilirubin	–
Endocrine function	Cortisol, TSH, fT3, fT4, AMH ^a^, FSH, LH, Estradiol ^b^, Glucose, Hb1Ac	TSH, fT4, fT3	–	AMH, FSH, LH, Estradiol Hb1Ac	TSH, fT4, fT3
Heart	ECG, CK, Troponin T and I Echocardiography^c^	CK, Troponin T and I^d^	–	CK, Troponin T^d^	–
Kidney	Creatinine, Urea, Urine dipstick	Creatinine, Urea	–	Creatinine, Urea	Urine dipstick
Fluid and electrolyte balance	Sodium, Potassium	Sodium, Potassium	–	Sodium, Potassium	–
Bone metabolism	Calcium, Albumin, Vitamin D_3_	–	–	–	Calcium, Albumin
Bone marrow	Complete blood count	–	Complete blood count	Complete blood count	–
Latent infections	Hepatitis B and C, HIV, Tuberculosis	–	–	–	–
Coagulation and fibrinolysis	INR, PT, PTT, D-Dimer, Fibrinogen, Ferritin	–	–	–	INR, PT, PTT
Gastrointestinal function	FC, C.diff, CMV^e^	–	–	–	–
Lung function	Spirometry and DLCO^f^	–	–	–	–
Neurological function	Neurological examination (cranial nerves and motoric and sensory function)	–	–	–	–

*ALP* alkaline phosphatase, *ALT* alanine aminotransferase, *AST* aspartate aminotransferase, *CK* creatine kinase, *DLCO* diffusing capacity for carbon monoxide, *ECG* electrocardiogram, *FC* fecal calprotectin, *FSH* follicle-stimulating hormone, *GGT* gamma-glutamyl transpeptidase, *INR* international normalized ratio, *LDH* lactate dehydrogenase, *LH* luteinizing hormone, *PT* prothrombin time, *PTT* partial thromboplastin time, *TSH* thyroid-stimulating hormone

^a^Female patients 18–42 years old

^b^Premenopausal female patients

^c^Patients at risk of developing ICI-related myocarditis (e.g., combination ICI, previous cardiotoxic therapies, cardiovascular diseases)

^d^During the first 4 doses before every ICI administration, thereafter every 3 doses

^e^Patients with preexisting diarrhea

^f^Patients with preexisting lung disease or radiotherapy to the lung or mediastinum

### Endocrine functions

Considering the high incidence of thyroid disorders (approximately 20%) [28] and hypophysitis (5.6–10.5%) [29] among patients receiving ICI, we recommend assessing fasting levels of cortisol, thyroid-stimulating hormone (TSH), as well as free T4 and T3 (fT4, fT3) [30] (Table 1) at baseline. TSH and fT3 and fT4 should be assessed at every ICI administration during the first 3 months and every 3–6 months thereafter.

Although the long-term consequences of ICIs on gonadal tissues are currently not well known [31], orchitis and epididymitis have been reported [32, 33]. We recommend assessing anti-Mullerian hormone (AMH) levels in female patients of childbearing potential (18–42 years) prior to ICI treatment initiation and every 3 months to determine the ovarian reserve since AMH correlates positively with fertility given it is secreted by vital follicles in the ovaries to ensure adequate counseling and treatment in reproductive-age patients [34]. Systematic assessment of sexual hormones (e.g., follicle-stimulating hormone [FSH], luteinizing hormone [LH] and estradiol in premenopausal female patients at baseline and every 3 months under ICIs could provide valuable real-world evidence regarding the risk of hypogonadism among reproductive-age patients [31]. Given the diurnal variations in hormone levels, the hormone measurements should be preferably done before 9 am.

Although the incidence of autoimmune adrenalitis under ICIs is low (0.9%) [35], ICI-induced hypophysitis often manifests with adrenal insufficiency due to adrenocorticotropic hormone (ACTH) deficiency. There are currently few data on the utility of serial cortisol measurements in asymptomatic patients. In a retrospective single-center analysis, 60% of the cortisol measurements in ICI patients were not interpretable because they were not sampled in the appropriate time window (i.e., 6–10 am), and only 36% (*n* = 8) of the patients with hypophysitis manifesting with ACTH deficiency were diagnosed with serial cortisol measurements [36]. As such, we recommend measuring cortisol levels during ICI treatment in case of symptoms of central or primary adrenal insufficiency (e.g., nausea, vomiting, fatigue, hyponatremia).

ICIs can induce insulin deficiency in about 2% of cases, presenting as new onset type 1 diabetes or worsening of preexisting type 2 diabetes [37]. Given that high dose corticosteroids are the cornerstone of the treatment of irAEs, it is important to recognize patients at risk of diabetes mellitus. We recommend assessing random glucose levels and Hb1Ac at baseline to exclude pre-diabetes prior to ICI treatment and doing Hb1Ac measurements every 3–6 months thereafter [30] (Table 1).

### Cardiac function

ICI-induced cardiotoxicity may involve all parts of the heart and is attributed to both inflammatory and non-inflammatory mechanisms. Inflammatory cardiotoxicity may manifest as myocarditis, peri-myocarditis, pericarditis, left ventricular dysfunction without myocarditis or coronary vasculitis [38, 39]. The incidence of ICI-related myocarditis is in the range of 1%, which is likely an underestimate [40]. Although cardiotoxicity is rare overall, a high case fatality rate (35–50%) of ICI-induced myocarditis has been reported [41, 42]. Detecting immune-related cardiac toxicities are critical because in most cases, they represent an absolute contraindication for continuing ICIs [42]. Among reported cardiac toxicities, ICI-induced myocarditis is particularly feared because of the risk of rapid fatal evolution [40–42]. The monitoring of ICI therapy remains challenging, as evidence-based guidelines are lacking. We suggest performing a clinical evaluation, an electrocardiogram, a measurement of serum troponin T and, where available, a troponin I at baseline in all patients prior to ICI treatment initiation and up to the 4th administration. If results are normal, these tests can be spaced to every three doses [43–45]. It is worth mentioning that troponin I and T are not expressed in the skeletal muscle of healthy adult patients, but troponin T is found within the fetal skeletal muscle. In case of immune-related myositis, which can be associated with myocarditis, the healing process of the skeletal muscle may lead to the expression of fetal isoforms of troponin T. In this setting, troponin T becomes less specific than troponin I, which remains cardiac specific [46, 47].

A baseline transthoracic echocardiography should also be discussed in all patients. In resource-limited settings, this test should be discussed in patients at high risk of developing ICI-induced myocarditis: patients treated with combined ICI therapy or with a combination of ICI therapy with another cardiotoxic therapy, patients with ICI-related non-cardiovascular events, prior cancer therapy-induced cardiac condition (e.g., anthracyclines, HER2-targeting molecules, and MEK1/2 inhibitors) [39], or cardiovascular disease [43, 48, 49].

The diagnosis of ICI-induced myocarditis is often challenging because of the wide range of potential clinical presentations. Having a baseline cardiological assessment facilitates the process of establishing the imputability of ICI when facing abnormal EKG, troponin dosage or echocardiography results during ICI therapy. In case of abnormal results at baseline, such as baseline troponin elevation, further explorations are required in collaboration with the cardio-oncology team in order to unveil an underlying cardiac condition. Although troponin is an organ-specific biomarker, it is not specific of myocarditis and several diagnoses should be considered: coronary artery disease, heart failure, arrhythmias, pulmonary embolism, cardiomyopathy, amyloidosis, hypertension, pulmonary hypertension, or arterial thromboembolism [50]. Thus, abnormal baseline troponin must prompt further investigation including at least a transthoracic echocardiography. Depending on the initial findings, further investigations may be performed (e.g., coronary angiogram, cardiac magnetic resonance imaging) without postponing ICI therapy initiation. Finally, in most cases, a preexisting cardiac condition should not contraindicate the introduction of ICI therapy.

### Musculoskeletal function

The reported incidence of rheumatic irAEs varies between 1.5 and 22% [51, 52]. Because of poor differentiation of irAE-related symptoms from other common cancer-related symptoms (e.g., pain or fatigue) as well as heterogeneous definitions and reporting of rheumatic irAEs, the true incidence may be underreported in clinical trials. ICIs can induce new onset rheumatic irAEs including life-threatening myositis, unmask rheumatoid arthritis (RA), and cause flares of preexisting rheumatic diseases [53]. While there are seropositive asymptomatic patients that will develop RA upon ICI treatment, this situation is rare and does not exclude these patients from ICI therapy. Most patients with rheumatic irAEs will not have detectable autoantibodies. Moreover, no intervention is currently available to prevent the onset of RA in these patients. We do not propose the assessment of rheumatic autoantibodies in asymptomatic patients prior to ICI initiation. However, elevations in CK or troponin T after initiation of ICI treatment could be a sign of myositis and should prompt further rheumatologic evaluation.

### Kidney function

The incidence of acute kidney injury (AKI) from ICIs, defined as an increase in serum creatinine of at least 1.5 times the baseline, was estimated to be 17% in a retrospective cohort study [50]. While acute interstitial nephritis (AIN) is found in most renal biopsy samples, acute tubular injury and other glomerular pathologies have been increasingly identified as potential aetiologies [54, 55]. A correct diagnosis is therefore crucial for adequate management of AKI. For example, in the case of acute tubular injury, *ex juvantibus* treatment with corticosteroids would not be beneficial but actually be harmful. Additionally, concomitant treatment with non-steroidal anti-inflammatory drugs (NSAIDs) and/or proton pump inhibitors as well as ICI combination treatments have been shown to be risk factors for the development of AIN. We recommend assessing creatinine and urea levels prior to ICI treatment and before every administration during the frist 3 months and every 4 to 6 weeks thereafter to estimate renal function (Table 1). A baseline urine dipstick should be obtained before starting treatment with ICIs, then at regular intervals (i.e., 3–6 months) to detect proteinuria and microhematuria. New onset sterile leukocyturia in the urine dipstick is a typical sign of AIN and the lack thereof suggests another aetiology.

### Fluid and electrolyte balance

In a meta-analysis of phase II and phase III randomized controlled trials, ICI treatment was associated with an increased risk of hyponatremia (relative risk [RR] 1.78, 95% confidence interval [CI] 1.12–2.80) and hypokalemia (RR 1.62, 95% CI 1.30–2.02) in patients with non-small cell lung cancer (NSCLC) [56]. Hypernatremia also occurs with corticosteroid or diuretic therapy (especially thiazides) and in patients with thyroid, adrenal, or pituitary disorders or hepatic, cardiac or renal insufficiency, as well as with syndrome of inappropriate antidiuretic hormone secretion (SIADH). Hyperkaliemia can be triggered by antihypertensive drugs (e.g., angiotensin-converting enzyme [ACE] inhibitors and sartans) as well as renal or adrenal insufficiency. We recommend obtaining sodium and potassium levels prior to ICI treatment initiation to detect preexisting disturbances and continuing to assess these values at every administration during the first 3 months and every 4–6 weeks thereafter (Table 1).

### Bone metabolism

Corticosteroids are applied in about 30–48% of patients with irAEs [57–60]. Long-term corticosteroid treatment in particular can increase the risk osteopenia and osteoporosis [61]. To mitigate the long-term risk of bone impairment, patients at risk should be screened for osteopenia/osteoporosis. In patients with known osteopenia or a history of osteoporosis, supplementation with vitamin D and calcium is recommended and bisphosphonate prevention therapy can be considered to reduce the risk of bone fractures [61, 62]. In addition, elevated or high-normal markers of bone resorption were found in a case series of 6 patients who developed new fractures or resorptive bone lesions under ICI treatment [63]. It is possible that ICIs negatively impact T-cell mediated skeletal remodeling, resulting in bone erosion or loss [63]. We suggest obtaining calcium and albumin levels at baseline and every 4–6 weeks (Table 1). Hypercalcemia can be a sign of bone metastases, thyroid disorders, or renal disease (e.g., tertiary hyperparathyroidism). Moreover, the treatment of bone metastases with bisphosphonates or denosumab as well as pancreatic and renal insufficiency or hypoparathyroidism (primary or ICI-induced) can lead to hypocalcemia [64]. Vitamin D_3_ status should be assessed before ICI treatment and patients with deficiency should initiate oral supplementation.

### Bone marrow function

Hematological irAEs are rare (3.6% of all grades, 0.7% of grade 3–4) but potentially life-threatening [65]. In particular, cytokine release syndrome with hemophagocytic syndrome, aplastic anemia, and pancytopenia is rare but potentially fatal irAEs [65]. Moreover, cancer patients often have alterations in one or all three blood cell lines, which can be due to previous oncological treatments (i.e., chemotherapy or radiotherapy) or be a consequence of cancer-related malnutrition and vitamin deficiency. Preexisting autoimmune or hematological disorders can also be accompanied by cytopenias. We suggest obtaining a complete blood count including hemoglobin, hematocrit, absolute count of red blood cells, leukocytes, and thrombocytes before the initiation of ICI treatment and prior to each administration of ICIs (Table 1) during the first 3 months and every 4–6 weeks thereafter.

#### Latent infections

It is important to identify patients with latent tuberculosis or viral infections. A latent infection not only influences the choice of immunosuppression for possible irAEs, but might require anti-infectious treatment depending on the level of immunosuppression. In agreement with other international guidelines [66], we recommend screening for viral hepatitis (HBsAg, total anti-Hbc, total anti-HCV), HIV (anti-HIV1/2, HIV ag p24), and tuberculosis (T-spot or Quantiferon test) at baseline.

### Coagulation and fibrinolysis

In a retrospective cohort study, 12% of lung cancer patients treated with ICIs developed diseases associated with disorders of the coagulation and fibrinolysis system [67]. It was also reported that T-cell activation strongly induces the production of tissue factor, a primary initiator of coagulation. This might explain the positive association between the occurrence of a coagulopathy and tumor response to treatment [67]. Hemostasis parameters (such as international normalized ratio [INR], prothrombin time [PT], partial thromboplastin time [PTT], D-dimer, and fibrinogen values) need to be determined at baseline to detect a coagulopathy under immunotherapy. Ideally, the level of ferritin should also be determined at baseline. Baseline ferritin levels may be elevated due to the underlying malignancy, metabolic syndrome, or renal insufficiency. In patients who develop fever, a marked increase in ferritin levels can indicate hemophagocytic lymphohistiocytosis [65, 68]. We recommend reassessing ferritin, D-dimer and fibrinogen only if a coagulopathy or hemophagocytosis is clinically suspected during treatment (Table 1) and INR, PT, and PTT every 3–6 months.

### Gastrointestinal function

Similar to other irAEs, the incidence of colitis is higher among patients receiving ICI combination treatment (32%) compared to those treated with anti-PD1/PDL1 antibodies alone (6%) [69]. It was shown that clinical assessment of diarrhea using the National Cancer Institute’s Common Terminology Criteria for Adverse Events (CTCAE) classification did not correlate well with the colitis severity as indicated by the duration of steroid therapy and requirement for additional immunosuppressive drugs (e.g., infliximab as indirect marker) [69]. In patients with immune-mediated diarrhea and colitis (IMDC), high fecal calprotectin (FC) levels correlated however well with active colitis and endoscopic inflammation [66]. In IMDC patients with endoscopic remission after immunosuppressive treatment, a significantly lower FC concentration was found at IMDC onset and after treatment, indicating that FC levels might be used as a biomarker to predict response to IMDC treatment. Among patients presenting with diarrhea prior to ICI treatment, measuring FC levels can be helpful to identify a preexisting inflammatory condition. Additionally, a stool culture and PCR for Clostridium difficile and Cytomegalovirus (CMV) should be performed to rule out an infectious cause [70]. We do not recommend the routine assessment of pancreas enzymes (e.g., lipase and amylase) given the lack of association between elevated enzymes and clinically manifest pancreatitis.

### Lung function

Although pulmonary toxicity is a rare ICI-associated complication, it has one of the highest death rates among all irAES [18]. An estimated 3.5% of all patients treated with ICIs develop relevant ICI-associated interstitial lung disease (ILD) [71]. Pneumonitis occurs earlier, more frequently, and is typically more severe in patients treated for lung cancer compared to other types of cancer [71]. Preexisting lung parenchymal diseases (such as emphysema and fibrosis) are risk factors for lung cancer [72], and impaired pulmonary function and dyspnea prior to initiation of ICIs are risk factors for ICI pneumonitis [73]. Therefore, pre-treatment consideration of pulmonary function and radiological lung parenchymal abnormalities are important firstly to estimate the risk of pulmonary toxicity and secondly to determine the evolution if ICI toxicity is suspected. Regardless of the cancer type, we recommend the assessment of pulmonary function by spirometry and measurement of diffusing capacity of the lung for carbon monoxide (DLCO) patients with preexisting lung disease (e.g., ILD, chronic obstructive pulmonary disease [COPD]) or previous radiotherapy of the lungs or mediastinum prior to ICI initiation (Table 1) given the increased risk of toxicity in lung cancer patients and the high prevalence of COPD and ILD in this population. However, we are aware that this type of functional testing might be difficult to implement in clinical routine. However, we encourage documentation of interstitial lung abnormalities detected on staging chest CT scans and pneumological evaluation in case of suspected pneumonitis under ICIs [72].

### Νeurological function

ICIs have been associated with several neurological irAEs, including paraneoplastic neurological syndromes (PNS) which can affect the central nervous system (e.g., encephalitis, myelitis), the peripheral nervous system (e.g., Guillain-Barré-syndrome, other neuropathies), the neuromuscular junction (e.g., myasthenia gravis, Lambert-Eaton-syndrome) and muscles (e.g., myositis, myopathy) [74]. Approximately 30–40% of cancer patients treated with neurotoxic chemotherapies (e.g., platinum-derivates and taxanes) develop chemotherapy-induced peripheral neuropathy (CIPN) [75]. Additionally, preexisting stable neuroimmunological conditions may potentially reactivate or worsen with ICI treatment [76].

The majority of cancer patients are elderly and neurological examinations in this age group frequently reveal abnormal results independently of a cancer diagnosis and treatment (e.g., due to subclinical polyneuropathy). It can be challenging to differentiate preexisting neurological conditions from PNS or irAEs that require different treatment that are critical for the patient’s outcome. We recommend a baseline clinical neurological examination including motoric and sensory function and cranial nerve status (Table 1). In patients with preexisting neurological symptoms, we suggest referral for a short electrophysiological screening (i.e., electroneurography to detect the preexisting extent of neuropathy) prior to ICI initiation. Findings from this screening might require additional laboratory or other evaluations (e.g., MRI cerebral and spinal, additional electrophysiology, EEG, CSF analysis).

## Conclusion

Consensus guidelines on the use of essential laboratory and functional tests prior to ICI initiation and at regular intervals during ICI treatment are lacking. Given the considerable variation in clinical practice in different countries, international interdisciplinary consensus guidelines are needed that follow a delphi process to reach true consensus with a sound methodological approach across subject experts. Our recommendations were developed for optimal early detection and management of irAEs with the minimal burden for patients and health care systems. In addition to different organ specialists, the inclusion of pharmacists and pharmacologists into the expert panel can facilitate the interpretation of abnormal test results which might be caused by ICI as well as other drugs co-administered during ICI treatment.

In addition to improving long-term outcomes and quality of life, this approach might even be cost-effective considering that the costs of laboratory tests are minimal compared to the costs of ICIs and hospitalization for the management of irAEs [77].

The systematic collection of a uniform data set in large patient cohorts might also enable future research and validation of diagnostic and prognostic biomarkers which ultimately facilitate early diagnosis and management of irAEs that contribute to a more favorable quality of life and survival in patients living with cancer [78].
